# Long-Term Effects of Childhood Cancer Treatment on Dentition and Oral Health: A Dentist Survey Study from the DCCSS LATER 2 Study

**DOI:** 10.3390/cancers13215264

**Published:** 2021-10-20

**Authors:** Juliette Stolze, Kim C. E. Vlaanderen, Frederique C. E. D. Holtbach, Jop C. Teepen, Leontien C. M. Kremer, Jacqueline J. Loonen, Eline van Dulmen-den Broeder, Marry M. van den Heuvel-Eibrink, Helena J. H. van der Pal, Birgitta Versluys, Margriet van der Heiden-van der Loo, Marloes Louwerens, Judith E. Raber-Durlacher, Dorine Bresters, Henk S. Brand

**Affiliations:** 1 Princess Máxima Center for Pediatric Oncology, 3584 CS Utrecht, The Netherlands; j.c.teepen@prinsesmaximacentrum.nl (J.C.T.); l.c.m.kremer@prinsesmaximacentrum.nl (L.C.M.K.); m.m.vandenheuvel-eibrink@prinsesmaximacentrum.nl (M.M.v.d.H.-E.); h.j.h.vanderpal@prinsesmaximacentrum.nl (H.J.H.v.d.P.); a.b.versluijs@prinsesmaximacentrum.nl (B.V.); m.vanderheiden@prinsesmaximacentrum.nl (M.v.d.H.-v.d.L.); d.bresters@prinsesmaximacentrum.nl (D.B.); 2 Academic Center for Dentistry Amsterdam (ACTA), Department of Oral Biochemistry, 1081 LA Amsterdam, The Netherlands; kcevlaanderen@gmail.com (K.C.E.V.); frederiqueholtbach@hotmail.com (F.C.E.D.H.); h.brand@acta.nl (H.S.B.); 3 Academic Center for Dentistry Amsterdam (ACTA), Department of Oral Medicine, 1081 LA Amsterdam, The Netherlands; judith@raber.nl; 4 Wilhelmina Children’s Hospital, University Medical Center Utrecht, 3584 EAUtrecht, The Netherlands; 5 Emma Children’s Hospital, Amsterdam UMC, University of Amsterdam, 1105 AZ Amsterdam, The Netherlands; 6 Radboud University Medical Center, 6525 GA Nijmegen, The Netherlands; Jacqueline.loonen@radboudumc.nl; 7 Emma Children’s Hospital, Amsterdam UMC, Vrije Universiteit Amsterdam, 1105 AZ Amsterdam, The Netherlands; eline.vandulmen-denbroeder@vumc.nl; 8 Willem-Alexander Children’s Hospital, Leiden University Medical Center, 2333 ZA Leiden, The Netherlands; m.louwerens@lumc.nl; 9 Department of Oral and Maxillofacial Surgery, Amsterdam University Medical Center, 1105 AZ Amsterdam, The Netherlands

**Keywords:** childhood cancer, late effects, dental developmental disorders, dental abnormalities, survivors, oral health

## Abstract

**Simple Summary:**

We aimed to identify the prevalence of and independent risk factors for dental and oral problems in childhood cancer survivors (CCSs). This cross-sectional study is part of the Dutch Childhood Cancer Survivor Study (DCCSS) LATER 2. Our study included survey data on 154 CCSs, on whom information from dentists on oral health data was received (71.3%). In total, 36.3% of survivors were reported to have at least one dental developmental disorder (DDD). The most prevalent DDDs were short-root anomaly (14.6%), agenesis (14.3%), and microdontia (13.6%). Risk factors for at least one DDD were younger age at diagnosis (<3 years vs. 5+ years) and dose-dependent alkylating agent therapy. This study provides more insight into risk factors for oral health problems in Dutch CCSs. This information is essential in order to improve early detection, prevention, and dental care of oral health problems in CCSs.

**Abstract:**

Objectives: The aim of this study was to identify the prevalence of and independent risk factors for long-term effects of childhood cancer treatment on the dentition and oral health in childhood cancer survivors (CCSs). Methods: This cross-sectional study is part of the Dutch Childhood Cancer Survivor Study (DCCSS) LATER 2. CCSs were diagnosed with cancer between 1963 and 2001. This study focuses on survey data of 154 CCSs on whom information about their oral health was received from their dentists (71.3%). Descriptive statistics and univariable and multivariable Poisson regression analyses were performed to determine the association between treatment characteristics and oral health data. Results: Of the study group, 36.3% had at least one DDD. The most prevalent DDDs were short-root anomaly (14.6%), agenesis (14.3%), and microdontia (13.6%). Risk factors for at least one DDD were younger age at diagnosis (<3 years) and dose-dependent alkylating agent therapy. Conclusions: This study provides more insight into risk factors for oral health problems in Dutch CCSs. This information is essential in order to improve early detection, prevention, dental care, and quality of life. Further studies are needed in order to better define dose-related radiotherapy exposure of the developing teeth in correlation with oral health problems.

## 1. Introduction

Over the past few decades, the survival rates for childhood cancer have increased considerably. Currently, almost 80% of children who are being treated for childhood cancer will survive more than 5 years after cancer diagnosis [1]. However, over 75% of childhood cancer survivors (CCSs) develop adverse late chronic health conditions arising from their former disease and its treatment [2,3]. Amongst these late effects, oral health problems related to cancer treatments have been reported [4]. Oral late effects may include dental developmental disorders, craniofacial abnormalities, gingivitis, dental caries, salivary gland dysfunction, and xerostomia [5,6,7,8,9,10,11,12].

Compared to healthy individuals, CCSs have a higher prevalence of oral problems [6,8,13,14,15,16]. Developmental disorders of the teeth in CCSs are often a result of cancer treatment during tooth development, due to the disruption of ameloblast and odontoblast activity [17]. In CCSs, a younger age at diagnosis [7,8,13,18,19], total body irradiation (TBI) or a higher dose of radiation exposure to the head and neck region [7,8,20], and treatment with a high dose of alkylating agents [8,21] or vinca alkaloids [22,23] were reported to be associated with increased risk for long-term oral problems and dental abnormalities. However, most of these studies were based on univariable comparisons, without adjusting for other potential risk factors. Reported dental developmental disorders (DDDs) include tooth agenesis, microdontia, enamel hypoplasia, arrested root development, delayed eruption, primary tooth retention, and taurodontism [6,7,8,14,24]. Awareness among dental professionals of these late effects is important, as long-term oral sequelae of chemotherapy and radiotherapy require long-term prevention and specialized dental care [25].

The present cross-sectional study was conducted in a well-defined Dutch national CCS cohort, and aimed to assess the prevalence of and independent risk factors for long-term effects of childhood cancer treatment on dentition and oral health. The major advantages of the present study are the large number of included participants, the long follow-up period of more than 15 years, comprehensive detailed oral health data obtained from the survivors’ own dentists, and investigation of the significance of clinically relevant risk factors.

## 2. Materials and Methods

This cross-sectional study is part of the so-called SALI subproject (SALI refers to hypoSALIvation, which is the main study objective). The SALI subproject is part of the Dutch Childhood Cancer Survivor Study (DCCSS) LATER 2. The SALI subproject was approved by the Medical Ethical Committee of the Amsterdam University Medical Center, the Netherlands (protocol number MEC2013_127). Informed consent was obtained from all participating subjects.

### 2.1. Participants

In the DCCSS LATER 2 study, CCSs were included from February 2016 until March 2020. Survivors were eligible for inclusion if they were diagnosed with childhood cancer between 1963 and 2001 in one of the 7 pediatric oncology centers in the Netherlands, aged 0–17 years at diagnosis of malignancy, and survived at least 5 years since diagnosis of the malignancy. This nationwide cohort of more than 6000 survivors will be described elsewhere (Teepen et al., manuscript submitted). In the SALI subproject, participants were included from three of the seven outpatient clinics of DCCSS LATER 2: Amsterdam University Medical Center (UMC) location VU, Leiden University Medical Center (LUMC), and Princess Máxima Center for Pediatric Oncology (PMC) in The Netherlands. An equal number of participants were invited for two study groups: CCSs who received H&N RT (including TBI), and CCSs who did not receive H&N RT.

### 2.2. Data Collection

Data with regard to gender, age at study, diagnosis, age at diagnosis, and treatment characteristics were collected by data managers using a uniform, standardized protocol. Participants in the SALI subproject were asked for permission to contact their dentist to request dental information from their dental files. After obtaining consent, a data extraction sheet was sent to the participant’s dentist. This data extraction sheet was designed to collect oral health data from patient records. The dentist was requested to return the data extraction sheet pseudonymized. If no response was received after a month, a reminder letter was sent. If still no response was received, after two weeks, a telephone reminder was conducted. The pseudonymized patient data were stored in the web-based Castor EDC data management system, which meets all legal requirements regarding Research Data Management and General Data Protection Regulation.

### 2.3. Statistical Analysis

Patient demographics and disease- and treatment-related characteristics were summarized using descriptive statistics and compared between the H&N RT and the non-H&N RT groups using the Mann–Whitney U test for continuous variables and Fisher’s exact test for categorical variables. Age at diagnosis was divided into three categories, based on age ranges described in previous papers on dental late effects: 0–2.99 years, 3–4.99 years, and ≥5 years [19,26]. Frequencies of DDDs and oral health data were reported and compared according to treatment modality and age at diagnosis using Fisher’s exact test. The association between orthodontic treatment and short-root anomaly was calculated using Fisher’s exact test. The distribution of teeth affected by different DDDs was reported using descriptive statistics. Multivariable logistic regression analyses were used to evaluate the association between potential risk factors and occurrence of DDDs. Because the outcome was common, and in such situations the odds ratios generated by logistic regression analyses overestimate the relative risk, we used Poisson regression models with log-link function and robust standard errors to calculate relative risks [27]. Potential risk factors were included in the multivariable model based on univariable analyses, previous studies [8,19,20], and clinical relevance, resulting in the variables gender, age at diagnosis, H&N RT, treatment with vinca alkaloids, treatment with epipodophyllotoxins, and the cyclophosphamide equivalent dose (CED) [28]. Additionally, we evaluated the contribution of other chemotherapy classes (anthracyclines, platinum compounds, and antimetabolites), but those were not included in the final model, as they were not significantly associated with DDDs in univariable analyses. Testing for trends of alkylating agent dose was based on the likelihood-ratio-based *p*-value for a model with the relevant continuous variable on the basis of exposed patients only. As the data collection in this multicenter study was standardized and controlled from one data center, with close cooperation between the outpatient clinics, no multilevel analyses were performed. IBM SPSS version 26.0 (IBM Corp. Armonk, NY, USA) was used to perform data analyses.

## 3. Results

### 3.1. Inclusion

A total of 306 of the 617 invited CCSs participated in the SALI subproject, of whom 216 (70.6%) gave permission to contact their dentist. Ultimately, 154 dentists (71.3%) returned the data extraction sheet. Figure S1 provides a flowchart of the inclusion process.

### 3.2. Patient Demographics and Treatment Characteristics

The total study sample comprised 154 survivors. Table 1 shows the characteristics of these survivors. There was an almost equal distribution between men (48.7%) and women (51.3%). The minimum time in years between diagnosis and enrollment of the study was 15.9 years, with a median time of 25.2 years. The median age at diagnosis was 5.2 years, with a range of 0.3–16.1 years. In the H&N RT group, 47 CCSs were included (30.5%), whereas the non-irradiated group consisted of 107 CCSs (69.5%). Additional data provided in Table S1 show the dose values (Gy) per field of H&N RT. Survivors who were treated with H&N RT had a significantly higher age at diagnosis (median 7.5 years) than survivors who did not receive H&N RT (median 4.0 years) (*p* = 0.002). A majority of the survivors were diagnosed with hematological malignancies (72.1%). Almost all survivors received chemotherapy (96.8%).

### 3.3. Oral Health Data

The data on oral health are presented in Table 2, stratified for treatment with/without H&N radiotherapy and for different age groups. According to their dentists, a majority of the CCSs had good oral hygiene (62.8%). Of all survivors, 20.4% and 10.7% were considered to have increased caries and periodontitis susceptibility, respectively. The prevalence of oral health problems did not differ significantly between survivors who received H&N RT or did not, except for a history of orthodontic treatment, which was higher among survivors who did not receive H&N RT versus those who did receive H&N RT (64.5% versus 37.0%).

### 3.4. Dental Developmental Disorders

**Figure 1:**
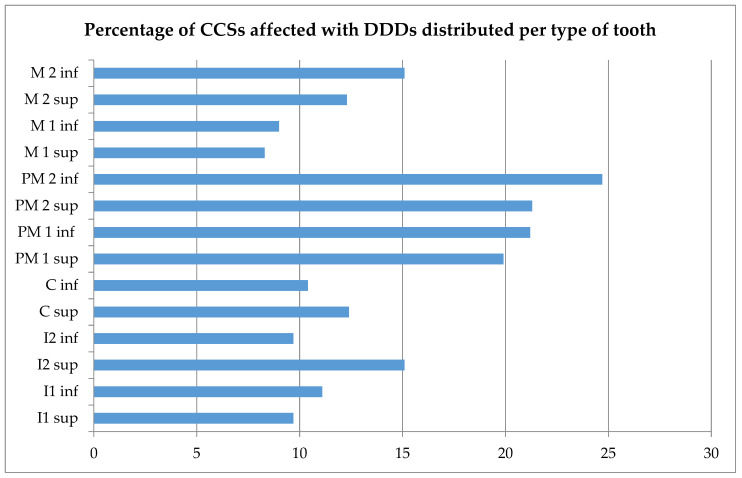
Percentages of Childhood Cancer Survivors (CCSs) affected by dental developmental disorders (DDDs), distributed per type of tooth. Sup: superior; inf: inferior; I1: central incisor; I2: lateral incisor; C: cuspid; PM1: first premolar; PM2: second premolar; M1: first molar; M2: second molar. Missing values were excluded from descriptive analysis.

In our study group, 36.1% had at least one DDD (Table 2). The most prevalent DDDs were short-root anomaly (14.6%), agenesis (14.3%), and microdontia (13.6%). More than 19.9% experienced a DDD in the lower or upper premolars (Figure 1). The distribution of teeth affected by different DDDs is provided in Figure S2. Agenetic teeth were mostly second premolars, while teeth affected by microdontia were mostly first premolars. All types of teeth were affected by short-root anomaly and hypomineralization. No significant association was found between short-root anomaly and a history of orthodontic treatment (*p* = 1.000). Table 3 shows the prevalence of DDDs stratified according to age at diagnosis.

### 3.5. Risk Factor Analysis

In univariable analysis, a significant association was found between ≥1 DDD and childhood cancer treatment with chemotherapy including alkylating agents (*p* = 0.019), and with chemotherapy including epipodophyllotoxins (*p* = 0.001). The prevalence of at least one DDD did not differ significantly between CCSs treated with TBI (53.3%) versus without TBI (34.1%), nor between CCSs treated with TBI (53.3%) versus H&N RT without TBI (26.7%) (*p* = 0.162 and *p* = 0.105, respectively). Among survivors who received H&N RT, those who received TBI had a significantly higher prevalence of microdontia (26.7%) than survivors who received H&N RT but not TBI (3.3%) (*p* = 0.036). Furthermore, CCSs who received TBI had an increased prevalence of short-root anomaly (35.7%) compared to CCSs who did not receive TBI (12.3%) (*p* = 0.034). A significant association was also found between short-root anomaly and childhood cancer treatment with chemotherapy including alkylating agents (*p* = 0.001).

We performed Poisson regression analysis to evaluate the possible role of patient- and treatment-related characteristics in the prevalence of 1 or more DDDs (Table 4). Gender was not associated with the risk of ≥1 DDD (RR, 1.03; 95% CI, 0.67 to 1.58), nor was H&N RT (RR, 1.15; 95% CI, 0.72 to 1.83), chemotherapy with epipodophyllotoxins (RR, 1.42; 95% CI, 0.91 to 2.22), or chemotherapy with vinca alkaloids (RR, 0.67; 95% CI, 0.41 to 1.09). Survivors younger than 3 years at diagnosis had a statistically significantly increased risk of developing a dental developmental disorder in comparison to survivors older than 5 years at diagnosis (>5 years versus <3 years, RR, 0.46; 95% CI, 0.27–0.78). Alkylating agent exposure was associated with a dose-dependent increased risk of DDD, with dose-tertile-specific RRs of 1.46 (95% CI, 0.78 to 2.73), 1.89 (95% CI, 1.03 to 3.47), and 2.61 (95% CI, 1.39 to 4.91), for >0–3999 mg/m^2^, 4000–9999 mg/m^2^, and ≥10,000 mg/m^2^, respectively (P_trend_ = 0.390).

## 4. Discussion

Based on detailed data of childhood cancer treatment and oral health information obtained via the survivors’ own dentists, this study provides important insights into the oral health in Dutch childhood cancer survivors. More than one third of CCSs included in this study experienced at least one DDD. The main risk factors for DDD were age at diagnosis—with a twofold increased risk for CCSs younger than 3 years vs. >5 years at diagnosis—and higher cumulative dose of alkylating agents, with a 2.6-fold increased risk for those treated with a dose ≥10,000 mg/m^2^.

### 4.1. Oral Health

It is difficult to disentangle the relationship between childhood cancer treatment and oral health, as oral health diseases are multifactorial; especially at older age, lifestyle, diet, oral hygiene, and salivary flow rates play an important role. In the present study, increased caries susceptibility was reported among 20.4% of the CCSs, which was not significantly different between CCSs who received H&N RT and those who did not. In another study among 5-year CCSs, significant positive correlations were found between the number of lesions in primary dentition and H&N RT and duration of chemotherapy, but not in permanent dentition [29].

Although our study did not include a group of healthy controls to compare results with, it is evident that the prevalence of DDDs in CCSs is increased. Prevalences in the current study were higher than reported prevalences in the general population for short-root anomaly (14.6% vs. 1–10% [30]) and tooth agenesis (14.3% vs. 4.6–6.3% [31]). Compared to a study among healthy Japanese high-school students, the prevalence of microdontia in the permanent dentition in our study was higher (13.6% vs. 7%), while the prevalence of peg-shaped teeth was comparable (3.4% vs. 2–3%) [32]. For peg-shaped teeth, the same teeth (second upper incisors and premolars) were affected among the Japanese high-school students as among CCSs in our study, which may indicate that this abnormality is not related to childhood cancer treatment. The prevalence of taurodontism (2%) in our study was lower compared to what was described in a review that reported a prevalence between 6% and 26% among CCSs [33]. Among our study group, premolars and second molars were most often affected by DDDs. Other studies have reported similar results [13,19,34]. For agenesis and microdontia, types of affected teeth were also similar [13,19,34].

Craniofacial abnormalities among CCSs have been reported by others [18]. Orthodontists reported that they postponed orthodontic treatment for 2 years after cessation of childhood cancer therapy [35]. In the present study, precautions in orthodontic treatment were not reported by any of the dentists, and only 16% of the dentists reported problems during or after the orthodontic treatment. As root resorption is known as a complication after orthodontic treatment [36], and no correlation was found between short-root anomaly and orthodontic treatment, the suggestion can be made that short-root anomaly might be associated with childhood cancer treatment, and not with the orthodontic treatment received afterwards.

### 4.2. Age at Diagnosis

Our study shows that younger age at cancer diagnosis (<3 years versus >5 years) is a risk factor for dental developmental disorders. This result is consistent with the assumption that dental developmental disorders are related to the stage of dental development and, thus, related to age at diagnosis and oncological treatment [37]. Tooth development comprises several stages, and the timing differs depending on the type of teeth [38]. Disturbance at different stages will lead to different developmental defects. At birth, mineralization of the first permanent molars begins. During the first year of life, mineralization of incisors and canines begins, followed by premolars and second molars in the second and third years of life, and ending with third molars between the eighth and eleventh years of life [39]. Age groups in this study were based on the schedule of tooth development [19,40]. In a study of 196 CCSs [19], a younger age at cancer diagnosis (<3 years) was also a significant, independent risk factor for severely abnormal Modified Dental Defect Index scores (MDDI: a single-index figure or classification representing the overall damage by dental developmental disturbances to permanent dentition [18,41]). In the present study, agenesis, microdontia, peg-shaped teeth, and persisting deciduous teeth were most prevalent in CCSs with an age of less than 3 years at diagnosis (*p* < 0.013). This is consistent with other studies that reported a significantly higher prevalence of agenesis, microdontia, and severe enamel hypoplasia among CCSs younger than 3 years at diagnosis [13,19,42]. Agenesis was also significantly more prevalent in CCSs younger than 5 years versus older than 5 years at ALL diagnosis [43]. In our study, short-root anomaly was prevalent in survivors of any age at diagnosis, and was not significantly different between the age at diagnosis categories, which is consistent with another study of 69 CCSs [44]. In contrast, in a study of 52 stem cell transplantation recipients, root development was mostly affected in the age group between 3 and 5 years at diagnosis [26].

### 4.3. Head and Neck Irradiation

In the present study, H&N RT did not contribute significantly to DDDs, which is consistent with other reports [19,21]. However, in one study there was a significant increase in the MDDI score with the maximum H&N RT dose within the youngest group (<3 years at diagnosis) [19]. Importantly, in our study sample, only 6 of 47 (12.8%) children aged < 3 years at diagnosis versus 34 of 47 (72.3%) children > 5 years at diagnosis received irradiation to the head and neck as part of their treatment. As a consequence, analyses on the effects of H&N RT were hampered by small numbers of CCSs receiving H&N RT.

Our study shows that CCSs who received TBI had an increased prevalence of microdontia as compared to CCSs who received other types of radiotherapy targeting the head and neck (*p* = 0.036). This is consistent with the results of a meta-analysis showing that survivors treated with TBI had an increased risk of developing microdontia compared to survivors who received cranial irradiation, but not TBI (*p* = 0.05) [7]. A possible explanation for a more severe adverse effect of TBI on dental development could be the higher fraction dose that was used during the time our survivors were treated. It is possible that higher fraction dose has a more detrimental effect irrespective of total cumulative irradiation dose. Further research is necessary in order to analyze a possible relationship between dental effects and fraction doses versus total cumulative irradiation doses.

A recent systematic review by Milgrom et al. presented data on dental developmental effects in CCSs who received RT to the head and neck, focusing on dose–volume parameters [20]. Risk factors included higher radiation dose to the developing teeth (>20 Gy) and a lower age at treatment. In the study of Kang et al. [19], in univariable analysis, a head and neck dose of ≥40 Gy influenced the severity of dental abnormalities in the youngest group, but not in the oldest group. In multivariate analysis, the effect of H&N RT was not dose-related; therefore, age seemed to be a more important risk factor. In the present study, mean cumulative irradiation doses are reported for radiation fields that were assumed to involve the teeth and oral cavity (Table S1). We chose not to include radiation doses in the regression analysis for several reasons: Firstly, we were not able to determine which fields—except for TBI—had definitely reached the jaws. Secondly, a minority of the CCSs who received H&N RT were younger than 3 years at diagnosis (12.8%) or between 3 and 5 years at diagnosis (14.9%)—the age groups for which we might expect a dose-dependent effect. Thirdly, H&N RT yes/no was not associated with DDDs in multivariable regression analyses.

### 4.4. Chemotherapy

The present study suggests that the use and dosage of alkylating agents is an important risk factor for dental developmental defects. As shown in animal studies, cyclophosphamide and other alkylating agents disturb the dentinogenesis process, in that agents bind to DNA in the S-phase of mitosis, resulting in early apoptosis [8]. Outcomes may reflect the stage of dental development at exposure to the alkylating agent, in such a way that, in the early stages, a greater effect on dentition is expected. Among 8522 CCSs treated before the age of 5 years, dose-dependent alkylating agent therapy (>4 g/m^2^) significantly increased the risk of one or more dental developmental disorder, independent of radiotherapy to the dentition [8]. Associations between usage and cumulative dose (>7.5 g/m^2^) of cyclophosphamide and dental abnormalities were also found in a study of 106 CCSs [45].

In univariable analysis, chemotherapy with epipodophyllotoxins was significantly associated with ≥1 DDD. However, in Poisson analysis, treatment with epipodophyllotoxins was not associated with the risk of ≥1 DDD. Until now, no studies had found a significant association between epipodophyllotoxins and dental developmental disorders. It has been suggested that vincristine and vinblastine disturb the microtubule calcium transport mechanism in ameloblasts [6,10], and may also interfere with the secretory function of ameloblasts and odontoblasts, disrupting collagen fibril formation and dentin matrix secretion [5,6,22,23]. However, we did not find vinca alkaloids to be a risk factor for DDDs, which was similar to results on dental abnormalities [8] and the individual defect index (IDeI) [16,21] in other studies.

### 4.5. Strengths and Limitations

A strength of this study was that we obtained data on oral health issues and dental developmental disorders from the survivors’ dentists instead of self-reported data from survivors, and that we received data from 71.3% of the approached dentists. Since at least 15 years had elapsed between cancer treatment and the retrieval of data from the dentists, numbers of oral health issues or dental developmental disorders might have been underreported, as not all requested data may have been retrievable from the patients’ records. As we did not have access to relevant radiographs, we were not able to carry out a more precise analysis of root defects such as tapered or blunted roots in addition to shortened roots. Therefore, we were not able to use the IDeI or MDDI indices for analysis, both of which are recommended indices for dental developmental disorders [41]. The SALI subproject selected CCS participants who had received H&N RT or TBI, and CCSs who had not received irradiation. Therefore, our study sample was not representative of the Dutch CCS cohort as a whole [46] (Teepen et al., manuscript submitted), as we invited an equal number of CCSs from two treatment groups. Therefore, the overall relevance of the outcomes should be interpreted with caution. However, we did not observe major differences in outcomes between the two groups. This selection procedure allowed us to properly investigate differences in DDDs between CCSs treated with H&N RT or TBI and those treated with chemotherapy only.

## 5. Conclusions

Dental developmental disorders are prevalent among CCSs. Age less than 3 years at diagnosis and dose-dependent alkylating agent therapy are the main risk factors for DDDs. Further studies are needed in order to better define dose-related radiotherapy exposure of the developing teeth in correlation with DDD.

This study provides essential information for oral health professionals and pediatric oncologists who assist in the detection, prevention, and appropriate dental care of dental developmental disorders, which can improve oral-health-related quality of life in childhood cancer survivors.

## Figures and Tables

**Table 1:** Patient and treatment related characteristics.

	Total *n* = 154 (100%)	H&N RT ^a^ *n* = 47 (30.5%)	No H&N RT *n* = 107 (69.5%)	*p*
Gender				
Male	75 (48.7)	27 (57.4)	48 (44.9)	0.165 *
Female	79 (51.3)	20 (42.6)	59 (55.1)	
Age at enrollment (years)	32.4 (16.8–56.6)	38.4 (21.1–56.6)	30.3 (16.8–51.6)	0.000 **
Age at cancer diagnosis (years)	5.2 (0.3–16.1)	7.5 (1.3–14.1)	4.0 (0.3–16.1)	0.002 **
0–2.99 (youngest)	46 (29.9)	6 (12.8)	40 (37.4)	
3–4.99 (middlest)	29 (18.8)	7 (14.9)	22 (20.6)	
>5 (oldest)	79 (51.3)	34 (72.3)	45 (42.1)	
Time since diagnosis (years) ^b^	25.2 (15.9–48.8)	31.0 (16.5–43.8)	24.4 (15.9–48.8)	0.000 **
0 < 20	30 (19.5)	5 (10.6)	25 (23.4)	
20 < 30	78 (50.6)	16 (34.0)	62 (57.9)	
≥30	46 (29.9)	26 (55.3)	20 (18.7)	
Diagnosis				
Hematological malignancy	111 (72.1)	36 (76.6)	75 (70.1)	0.442 *
Brain tumor	7 (4.5)	7 (14.9)	0 (0.0)	0.000 *
Solid tumor	36 (23.4)	4 (8.5)	32 (29.9)	0.004 *
Type of treatment				
H&N RT		47 (100)		
Head/cranium		26 (55.3)		
Brain		21 (44.7)		
Face		5 (10.6)		
Neck ^c^		5 (10.6)		
TBI (HSCT) ^d^		16 (34.0)		
Chemotherapy	149 (96.8)	42 (89.4)	107 (100.0)	
Alkylating agents ^e^	101 (65.6)	33 (70.2)	68 (63.6)	0.466 *
Vinca alkaloids ^f^	129 (83.8)	34 (72.3)	95 (88.8)	0.017 *
Chemotherapy only			58 (54.2)	
Chemotherapy and RT but no H&N			12 (11.2)	
Chemotherapy and HSCT without RT ^g^			8 (7.5)	
Chemotherapy and surgery without RT			29 (27.1)	

Values are presented as *n* (column%) or median (range). Numbers do not always add up to 100%, because of rounding. H&N RT: head/neck radiotherapy; TBI: total-body irradiation; HSCT: hematopoietic stem cell transplantation. ^a^ Patients treated with H&N RT and/or TBI. ^b^ At enrollment of the study. ^c^ Of the 5 patients, 1 received autologous HSCT and 1 allogeneic HSCT. ^d^ Of the 16 patients, 4 received autologous HSCT and 12 allogeneic HSCT. ^e^ Including cyclophosphamide, ifosfamide, busulfan, and melphalan. ^f^ Including vincristine and vinblastine. ^g^ One patient received autologous HSCT and seven allogeneic HSCT. * Fisher’s exact test. ** Mann–Whitney U test.

**Table 2:** Distribution of oral health data by type of treatment and age at diagnosis, as assessed by dentists.

		Response (%) ^a^	Total *n*(%) ^b^	H&N RT				No H&N RT				
				0–3 years, *n*	3–5 years, *n*	> 5 years, *n*	Total, *n*(%) ^b^	0–3 years, *n*	3–5 years, *n*	> 5 years, *n*	Total, *n*(%) ^b^	*p* ^e^
Oral health												
	Poor oral hygiene	96.1	7 (4.7)	0	1	1	2 (4.4)	4	1	0	5 (4.9)	1.000
	Moderate oral hygiene	96.1	48 (32.4)	1	1	14	16 (35.6)	11	6	15	32 (31.1)	0.706
	Good oral hygiene	96.1	93 (62.8)	5	4	18	27 (60.0)	25	13	28	66 (64.1)	0.721
	Increased caries susceptibility	92.2	29 (20.4)	0	0	11	11 (26.2)	7	1	10	18 (18.0)	0.361
	High susceptibility to developing periodontal problems	90.9	15 (10.7)	0	0	6	6 (14.6)	4	2	3	9 (9.1)	0.373
Oral health problems												
	Trismus	88.3	2 (1.5)	0	0	0	0 (0)	2	0	0	2 (2.1)	1.000
	TMD	85.7	6 (4.5)	0	0	1	1 (2.5)	1	1	3	5 (5.4)	0.667
	Xerostomia	80.5	5 (4.0)	0	0	2	2 (5.4)	0	0	3	3 (3.4)	0.634
	Hyposalivation	83.1	6 (4.7)	0	0	2	2 (5.1)	1	0	3	4 (4.5)	1.000
	Complaints of altered taste	72.7	2 (1.8)	0	0	0	0 (0)	0	1	1	2 (2.5)	1.000
	Fungal infection	82.5	1 (0.8)	0	0	0	0 (0)	0	0	1	1 (1.1)	1.000
	Generalized severe tooth wear	92.2	2 (1.4)	0	0	1	1 (2.2)	0	1	0	1 (1.0)	0.535
	Leukoplakia	91.6	0 (0)	0	0	0	0 (0)	0	0	0	0 (0)	-
	Squamous-cell carcinoma	90.3	0 (0)	0	0	0	0 (0)	0	0	0	0 (0)	-
	Other abnormalities	87.7	17 (12.6)	1	3	4	8 (18.6)	3	1	5	9 (9.8)	0.170
Orthodontic												
	Craniofacial growth disorders	84.4	5 (3.8)	0	0	2	2 (4.8)	1	0	2	3 (3.4)	0.658
	Malocclusion	86.4	17 (12.8)	0	1	4	5 (11.6)	5	0	7	12 (13.3)	1.000
	History of OT	66.9	59 (57.3)	2	3	5	10 (37.0)	23	13	13	49 (64.5)	0.023
	Precautions in OT	66.1	0 (0)	0	0	0	0 (0)	0	0	0	0 (0)	-
	Problems in OT	62.7	6 (16.2)	0	0	2	2 (28.6)	1	0	3	4 (13.3)	0.315
Dental developmental disorder												
	Agenesis ^c^	95.5	21 (14.3)	1	0	3	4 (8.9)	11	3	3	17 (16.7)	0.307
	Microdontia	95.5	20 (13.6)	1	1	3	5 (11.1)	12	2	1	15 (14.7)	0.794
	Peg-shaped teeth	95.5	5 (3.4)	0	0	0	0 (0)	5	0	0	5 (4.9)	0.324
	Hypomineralization	94.8	9 (6.2)	0	0	3	3 (6.7)	4	0	2	6 (5.9)	1.000
	Taurodontism	95.5	3 (2.0)	0	0	1	1 (2.2)	0	1	1	2 (2.0)	1.000
	Short-root anomaly	93.5	21 (14.6)	1	3	6	10 (23.3)	4	2	5	11 (10.9)	0.071
	Persisting deciduous teeth	94.8	7 (4.8)	1	0	0	1 (2.2)	4	2	0	6 (5.9)	0.438
Number of dental developmental disorders ^d^		95.5										
	0		94 (63.9)	3	3	23	29 (64.4)	17	14	34	65 (63.7)	
	≥ 1		53 (36.1)	2	3	11	16 (35.6)	21	8	8	37 (36.3)	1.000

TMD: temporomandibular dysfunction; OT: orthodontic treatment. Values are presented as *n* (column %) of dentists who answered ‘yes’ on the item. ^a^ Response (%) shows the percentage of survivors for whom information on this specific outcome was provided by their respective dentist. ^b^ Missing values were excluded from descriptive analysis. ^c^ Cases with agenesis of third molars were excluded. ^d^ In case a value of one of the seven DDDs was missing, the relevant DDD was defined as absent (5 cases). If more than one variable was missing, the sum of DDDs was excluded from this analysis (7 cases). ^e^ Fisher’s exact test (H&N RT vs. non-H&N RT).

**Table 3:** Prevalence of dental developmental disorders according to age at diagnosis.

	Age at diagnosis ^a^				
	0–3 years ^b^	3–5 years ^b^	> 5 years ^b^	Total ^b^	*p* ^c^
Agenesis	12 (27.9)	3 (10.7)	6 (7.9)	21 (14.3)	0.013
Microdontia	13 (30.2)	3 (10.7)	4 (5.3)	20 (13.6)	0.001
Peg-shaped teeth	5 (11.6)	0 (0.0)	0 (0.0)	5 (3.4)	0.004
Hypomineralization	4 (9.3)	0 (0.0)	5 (6.6)	9 (6.2)	0.272
Taurodontism	0 (0.0)	1 (3.6)	2 (2.6)	3 (2.0)	0.587
Short-root anomaly	5 (11.6)	5 (18.5)	11 (14.9)	21 (14.6)	0.736
Persisting deciduous teeth	5 (11.9)	2 (7.1)	0 (0.0)	7 (4.8)	0.006
≥1 DDD	23 (53.5)	11 (39.3)	19 (25.0)	53 (36.1)	0.008

DDD: dental developmental disorder. ^a^ Missing values were excluded from descriptive analysis. ^b^ Values are presented as *n* (valid %) of dentists who answered ‘yes’ on the item in the relevant age group. ^c^ Fisher’s exact test.

**Table 4:** Poisson regression analysis for dental developmental disorders in childhood cancer survivors (*n* = 145).

Variable	Number of Survivors §	≥1 DDD, *n*	Relative Risk	95% CI	*p*
Gender					
Male	72	26	1.0 (ref)		
Female	73	27	1.03	0.67 to 1.58	0.896
Age at diagnosis					
0 < 3 years	42	23	1.0 (ref)		
3 < 5 years	28	11	0.79	0.45 to 1.38	0.398
>5 years	75	19	0.46	0.27 to 0.78	0.004
Type of treatment					
No H&N RT	101	37	1.0 (ref)		
H&N RT	44	16	1.15	0.72 to 1.83	0.561
Chemotherapy					
No vinca alkaloids	25	12	1.0 (ref)		
Vinca alkaloids	120	41	0.67	0.41 to 1.09	0.107
No Epipodophyllotoxins	108	31	1.0 (ref)		
Epipodophyllotoxins	37	22	1.42	0.91 to 2.22	0.122
CED, mg/m^2^ *					
No alkylating agents	52	12	1.0 (ref)		
<4000	42	13	1.46	0.78 to 2.73	0.237
4000–9999	31	15	1.89	1.03 to 3.47	0.040
≥10,000	20	13	2.61	1.39 to 4.91	0.003

Dental developmental disorders include agenesis, microdontia, peg-shaped teeth, hypomineralization, taurodontism, short-root anomaly, and persisting deciduous teeth. Abbreviations—DDD: dental developmental disorders; H&N RT: head and neck radiotherapy; CED: cyclophosphamide equivalent dose [28]; (ref): reference category. § Numbers do not always add up to the total, because of missing values. * Categories were based on approximate tertiles among exposed patients with ≥ 1 dental developmental disorder.

## Data Availability

The data that support the findings of this study are available on request from the corresponding author.

## Supplementary Materials

The following are available online at https://www.mdpi.com/article/10.3390/cancers13215264/s1: Table S1: Dose values per field of H&N RT; Figure S1: Flowchart of the inclusion process; Figure S2: Distribution of teeth affected by different dental developmental disorders in childhood cancer survivors.
