# Supplementary material for: Long-Term Effects of Childhood Cancer Treatment on Dentition and Oral Health: A Dentist Survey Study from the DCCSS LATER 2 Study
Source: Cancers (Basel). 2021 Oct 20;13(21):5264. doi: 10.3390/cancers13215264 (PMC8582458; doi:10.3390/cancers13215264)
Supplement: Supplementary file 1 [file cancers-13-05264-s001.zip › cancers-1383026-supplementary.pdf]

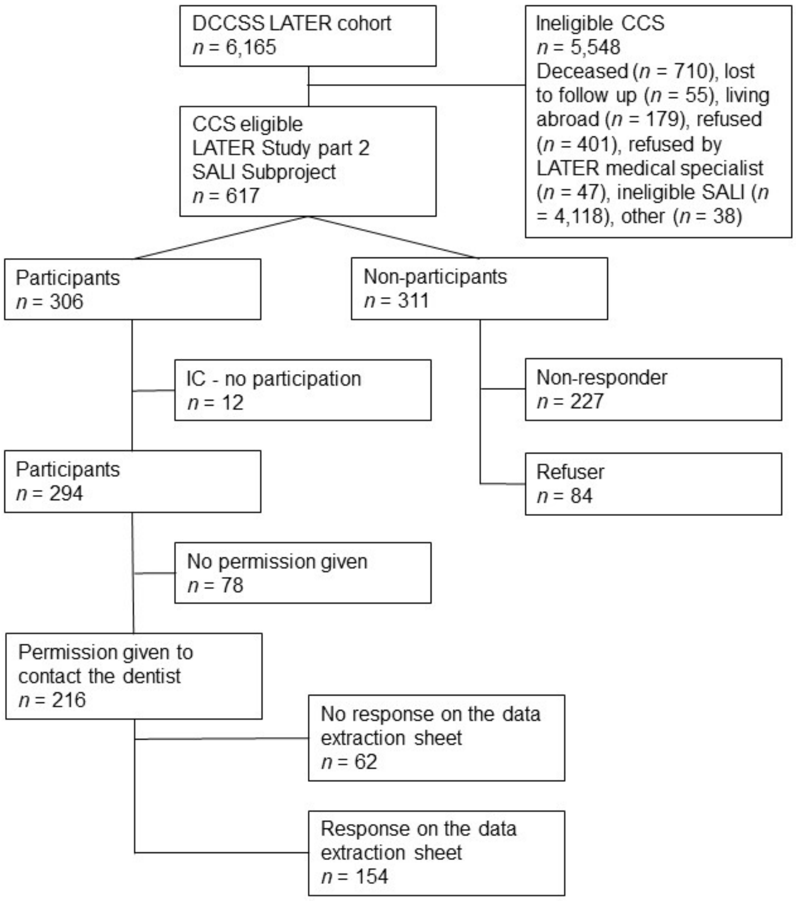

Figure S1. Flowchart of the inclusion process
DCCSS LATER; Dutch Childhood Cancer Survivor Late Effect Study, IC – no participation; survivors signed informed consent for participation however data collection was hampered for different reasons

Table S1. Dose values per field of H&N RT

	**Total cumulative irradiation dose (Gy)**	
	**Mean (sd)**	**Median (range)**
H&N RT (*n* = 47)	26.41 (19.24)	25.00 (7.50–100.80)
Head/cranium		
Brain (*n* = 21)	33.03 (13.27)	25.00 (18.00–60.00)
Face (*n* = 5)	51.08 (32.18)	39.60 (25.00–100.80)
Neck (*n* = 5)	29.84 (9.90)	30.00 (19.80–40.00)
TBI (*n* = 16)	8.94 (2.14)	7.50 (7.50–12.00)

Gy; gray, H&N RT; head/neck radiotherapy, TBI; total body irradiation

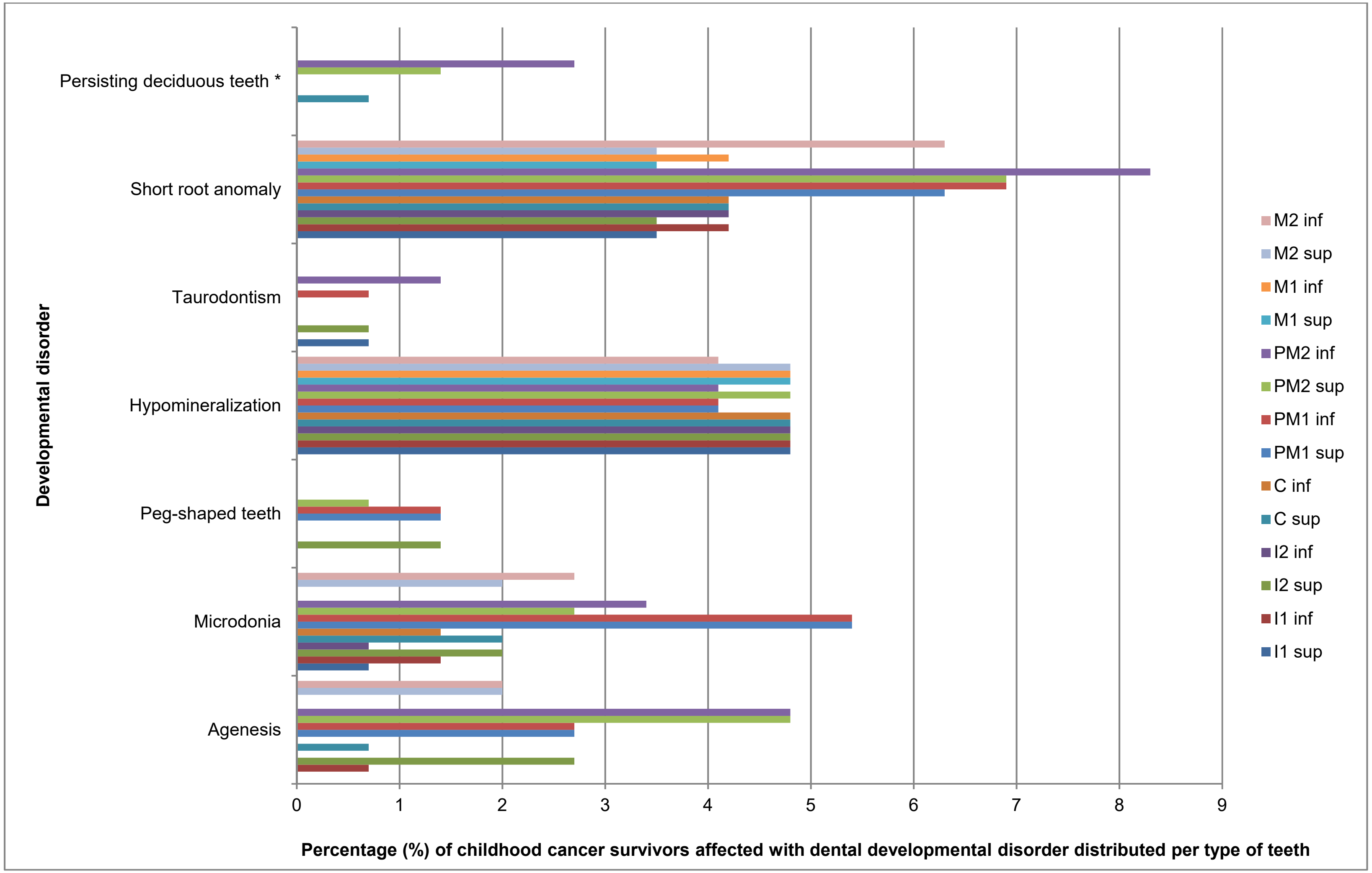

Figure S2. Distribution of teeth affected by different dental developmental disorders in childhood cancer survivors.
Sup; superior, inf; inferior, I1; central incisor, I2; lateral incisor, C; cuspid, PM1; first premolar, PM2; second premolar, M1; first molar, M2; second molar. Missing values were excluded from descriptive analysis. *relevant elements should be read as primary teeth.
